# Short-Interval Sequential CAR-T Cell Infusion May Enhance Prior CAR-T Cell Expansion to Augment Anti-Lymphoma Response in B-NHL

**DOI:** 10.3389/fonc.2021.640166

**Published:** 2021-06-30

**Authors:** Yuan Meng, Biping Deng, Luan Rong, Chuo Li, Weiliang Song, Zhuojun Ling, Jinlong Xu, Jiajia Duan, Zelin Wang, Alex H. Chang, Xiaoming Feng, Xiujuan Xiong, Xiaoli Chen, Jing Pan

**Affiliations:** ^1^ State Key Laboratory of Experimental Hematology, National Clinical Research Center for Blood Diseases, Institute of Hematology & Blood Diseases Hospital, Chinese Academy of Medical Sciences & Peking Union Medical College, Tianjin, China; ^2^ Cytology Laboratory, Beijing Boren Hospital, Beijing, China; ^3^ Department of Hematology, Beijing Boren Hospital, Beijing, China; ^4^ Clinical Translational Research Center, Tongji University School of Medicine, Shanghai, China; ^5^ Department of Pathology, Basic Medical College of Nanchang University, Nanchang, China; ^6^ Ganzhou Key Laboratory of Molecular Medicine, the Affiliated Ganzhou Hospital of Nanchang University, Ganzhou, China; ^7^ State Key Laboratory of Experimental Hematology, Boren Clinical Translational Center, Department of Hematology, Beijing Boren Hospital, Beijing, China

**Keywords:** B-NHL, CAR-T, CD19, CD22, CD20

## Abstract

Chimeric antigen receptor (CAR)-T cell therapy emerges as a new treatment for refractory or relapsed (r/r) B-cell non-Hodgkin lymphoma (B-NHL); however, the overall response rate (ORR) of which in the B-NHL patients is much lower compared to the patients with r/r B acute lymphoblastic leukemia (B-ALL). We previously confirmed that sequential infusions of CD20 and CD22 CAR-T cells significantly improved the prognosis of the B-NHL patients, while some advanced patients still progressed to death during these CAR-T cell treatments. In this study, we showed that timely sequential administration of the second CAR-T cells could enhance expansion of prior CAR-T cells with stronger tumor-killing capacity *in vitro* and *in vivo*. We further conducted compassionate treatments on two advanced B-NHL patients with short-interval sequential infusions of CD19/22/20 CAR-T cells. Disease progression was observed in both patients after primary CAR-T cell infusion but robust re-expansion of prior CAR-T cells and anti-tumor effects was induced by infusion of a secondary CAR-T cells. These results indicate sequential infusions of CAR-T cells with a short interval may improve therapeutic efficacy in the B-NHL patients by promoting expansion of prior CAR-T cells.

## Introduction

Aggressive B-cell lymphomas encompass a heterogeneous group of diseases, including diffuse large B-cell (DLBCL) and Burkitt lymphoma (BL) (1). As a highly aggressive B-cell non-Hodgkin lymphoma (B-NHL) (2), BL accounts for a majority of B-NHL in children in the developed world (1). Despite intensive chemotherapy agents (1) as well as targeting therapy of rituximab (3), the prognosis for the patients with relapsed/refractory (r/r) BL remains dismal, and the average overall survival rate is 25% or lower (4). In recent years, many clinical trials were conducted to evaluate therapy effects of CD19 Chimeric antigen receptor (CAR) T cells on r/r B-cell lymphoma, mainly r/r DLBCL. However, the response rate is much lower than that in r/r B-ALL patients with the overall response rate (ORR) and complete remission rate (CR) varying from 53 to 82% and 40 to 63%, respectively (5–8).

Our previous sequential CD19-22-20 CAR-T cell therapies demonstrated 41.7% overall CR rate in 17 pediatric patients with r/r B-cell lymphoma, including 13 BL (9). For the non-remission patients who received the prior CD19 CAR-T cells, CD20 and CD22 CAR-T cells were subsequently infused on day 30, and three of six patients further achieved CR. With extended duration of CAR-T cell expansion which was contributed by separated expansion of each kind of CAR-T cells, 70.6% overall CR rate was further achieved in 6 months after traditional sequential CAR-T cell therapies. However, CD19 CAR-T cells were usually undetectable by flow cytometry (FCM) in peripheral blood (PB) in our center after infusion for one month. *In vivo* expansion and persistence of CAR-T cells has been shown to contribute to clinical prognosis in hematological malignancies, and particularly in lymphomas (10–12). Rapid CAR-T cell exhaustion was often observed in advanced patients after CD19 CAR-T cell infusion, and it seemed tough for traditional CAR-T cell therapy to prevent disease aggravation. Recently, the cocktail CD19/22 CAR-T cell therapies improved the outcome of aggressive B-cell lymphoma with 72.2% ORR and 50% CR rate, in which the CAR-T cells with different targets contributed to enhanced antitumor effects (13), while there were mainly DLBCL patients and few BL patients enrolled.

We hypothesize that sequential CAR-T cell infusions may induce co-expansion of different CAR-T cells when residual prior CAR-T cells still remain detectable in PB, which leads to the prolonged duration of peak expansion of CAR-T cells with enhanced antitumor effects. That is, sequential infusions of different CAR-T cells with short interval augmented CAR-T cells and enhanced anti-tumor effects, which is consistent with the synergistic effects of multi-agent immunotherapies on eradicating disease and prolonging remission in the patients with relapsed hematologic malignancies (14). Validation experiments *in vitro* and in animal model were conducted first, and we hypothesize that such short-interval sequential CAR-T infusion was critical in therapeutic outcomes observed in two advanced B-cell lymphomas.

## Methods

### Cell Line

The Raji cell line was purchased from the American Type Culture Collection (ATCC, Manassas, VA, USA) and routinely cultured in RPMI-1640 medium (Thermo Fisher Scientific, Waltham, MA, USA) containing 10% fetal bovine serum (FBS; Thermo Fisher Scientific), 100 U/ml penicillin and 100 µg/ml streptomycin (Invitrogen, USA) in 5% CO_2_ at 37°C.

### Flow Cytometric Immunophenotyping

The following antibodies were used for FCM based immunophenotype detection: FITC: anti-CD20, anti-CD38, anti-CD15, anti-HLA-DR, anti-CD9, anti-CD7, anti-IgG1, anti-Kappa, anti-IgM, anti-cKi67; PE: anti-CD22, anti-CD34, anti-CD10, anti-CD13, anti-CD81, anti-CD123, anti-IgG1, anti-Lambda; PerCP: anti-IgG1, anti-CD45; APC: anti-CD19 (all BD Pharmingen, San Diego, California). For the staining preparation, red blood cells were lysed, and white blood cells were calculated and resuspended in phosphate-buffered saline (PBS) with 2% fetal bovine serum. Samples were analyzed on FACSCalibur, and the collected data were analyzed by FlowJo software (version 10).

### Construction and Detection of CAR

Lentiviral vectors carrying second generation anti-CD19, anti-CD20 or anti-CD22 CAR (YK-CD22BB-002) with 4-1BB co-stimulatory and CD3 signaling domains were constructed as previously described (15, 16). Briefly, the CD19 recognition domain was composed of a single-chain fragment variable (scFv) region derived from the FMC63 monoclonal antibody. The CD20 and CD22 recognition domain were composed of scFv regions obtained from human antibody phage display library. CD19 or CD22 CAR-T cell expansion can be distinguished by FCM with proprietary specific CD19 or CD22 CAR-T cell detection reagent (CD19-CAR-Green and CD22-CAR-Green, respectively from Shanghai YaKe Biotechnology Ltd., Shanghai, China). CD20 CAR-T cells were detected by FCM using biotinylated human IgG Fab fragment (Jackson ImmunoResearch Laboratories, West Grove, Pennsylvania) as the first antibody, and Streptavidin conjugated with APC (BD Pharmingen, San Diego, California) for CAR detection.

### Manufacture of CAR-T cells

Peripheral blood mononuclear cells (PBMCs) collected from patients were stimulated with magnetic beads coated with anti-CD3/CD28 antibodies (Life Technologies, Carlsbad, CA, USA; now owned by Thermo Fisher Scientific, Waltham, MA, USA) overnight. The next day, transduction was performed at multiplicity of infection 1:10 ratio. Transduced cells were cultured in X-VIVO 15, a serum-free medium (Lonza) with 300 IU/ml interleukin-2 (IL-2) (Sigma-Aldrich, St. Louis, MO, USA). Transduction efficiency was defined as the ratio of CAR-T to CD3^+^ T cells, determined by FCM. Cell viability was determined by Trypan blue (Sigma-Aldrich, St. Louis, MO, USA) exclusion.

### Expansion and Tumor-Killing Capacity of CAR-T Cells *In Vitro*

Different treatments were tested to determine an appropriate expansion model at 3:1, 1:1 or 1:3 ratio of effector-to-target (E:T). In first group, CAR-T cells were coated with anti-CD3/CD28 beads. In second group, Raji cells were pre-treated with 10 ug/ml Mitomycin (MMC-Raji). And the third group was administrated with blank treatment. CD19 CAR-T cells were alone as the control group. Group 3 in which both CAR-T cells and Raji cells were not pre-treated was chosen for following experiments. Prior CD19 CAR-T cells were labeled by CellTrace CFSE (Thermo Fisher Scientific, Waltham, MA, USA) and co-cultured with Raji cells for 24 h, then the subsequent CD19, CD22 and T cells transduced with pCDH empty vector (pCDH) were labeled with CellTrace Violet (Thermo Fisher Scientific, Waltham, MA, USA) and were subsequently added to the culture system respectively, as well as media for the control group. The absolute number of prior CD19 CAR-T cells labeled CFSE and live Raji cells was counted at several time points by FCM using Countbright counting beads (Thermo Fisher Scientific, Waltham, MA, USA) to evaluate the effect of the residual CD19 CAR-T cells expansion as well as tumor-killing capacity by sequentially infused CAR-T cells. Specific Lysis % was calculated as 100 × [(experimental killing − spontaneous killing)/(maximal killing − spontaneous killing)].

### Expansion and Tumor-Killing Capacity of CAR-T Cells *In Vivo*

Groups of 5 to 9-week-old female NSG mice (NOD-Prkdcscid Il2rgtm1/Bcgen) (Biocytogen, Beijing, China) were injected 1 × 106 luciferase+ Raji cells *via* tail vein on day 0. Tumor burden was measured using XenogenIVIS-200 Spectrum camera in mice that have been anesthetized and injected intraperitoneally with 3 mg D-luciferin (YEASEN, Shanghai, China) using Xenogen IVIS-200 Spectrum system (Caliper Life Sciences, USA). The bioluminescent signals were analyzed using Living Image Version 4.1 software (Caliper Life Sciences, Hopkinton, MA) as photons/sec/cm2/sr. CD19 CAR-T cells in peripheral blood of the transplanted mice were detected using a recombinant human CD19-Fc chimera protein (R&D Systems, Minneapolis, USA) and a secondary anti-human IgG Fc-APC antibody (BioLegend, San Diego, USA).

### Patients and Clinical Procedure

We conducted compassionate treatments in two patients with advanced B-cell malignancies who were heavily treated and refractory to chemotherapies. Due to advanced stage and severe complications, they were excluded from clinical trials. The treatments were approved by the Institutional Review Board (IRB) of Beijing Boren Hospital, and informed consent was obtained in accordance with the Declaration of Helsinki. Both patients matched the diagnostic criteria for r/r B-cell lymphoma according to National Comprehensive Cancer Network (NCCN) clinical practice guidelines in oncology: B-cell lymphoma, version 4.2019 (17). Immunotherapeutic targets were confirmed by immunohistochemistry **(**IHC) and FCM. Sequential administration of different CAR-T cells was given by intravenous injection, and expansion of different CAR-T Cell in PB was monitored by FCM. Response was evaluated by image tests such as computed tomography (CT) scan and IHC accordance to NCCN clinical practice guidelines in oncology (18). Cytokine release syndrome (CRS) and Immune effector cell-associated neurotoxicity syndrome (ICANS) was assessed and intervened according to American Society for Transplantation and Cellular Therapy (ASTCT) CRS, ICANS consensus Grading system and NCCN guideline for management of immunotherapy-related toxicities (8, 19, 20).

These patients were treated by modified sequential infusions of CD19-22-20 CAR-T cells. CAR-T cells were manufactured from PBMCs collected by leukapheresis and frozen for multiple uses. Before each CAR-T Cell infusion (day 0), the patients received lymphodepleting chemotherapy with Fludarabine (30 mg/m^2^/day) and Cyclophosphamide (250 mg/m^2^/day) on days -5 to -3. Bridging chemotherapies were given for lymphodepletion when they had progressive progression. The time spots of sequential CAR-T cell infusions depended on the expansion of prior CAR-T cells and disease status.

### Statistical Analysis

The results were presented as means ± SEM. The unpaired t-test was used to determine the statistical significance for comparisons of two or more groups. Statistical analyses were performed using GraphPad Prism software (La Jolla, CA, USA). P <0.05 was considered statistically significant (*P <0.05, **P <0.01, ***P <0.001).Results

### Sequential Administration of CAR-T Cells Amplified the Prior CD19 CAR-T cells *In Vitro*

**Figure 1:**
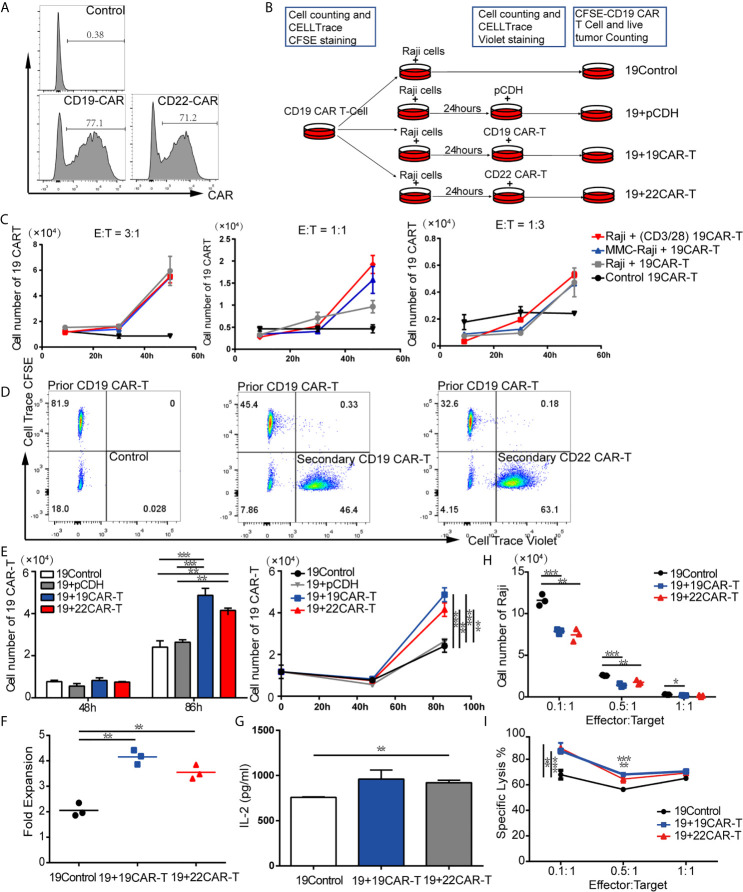
Sequential administration of CAR-T cells amplified the prior CD19 CAR-T cells *in vitro.* **(A)** Transduction efficiency of CD19 and CD22 CAR-T. **(B)** Experimental procedure of expansion models *in vitro*. **(C)** Expansion curves at three ratios of effector-to-target (E:T) under different treatment. **(D)** Flow cytometry (FCM) analysis of prior CD19 CAR-T cells labeled CellTrace CFSE and subsequent 19 CAR-T cells or 22 CAR-T cells labeled CellTrace Violet. **(E)** Absolute cell number (upper left panel), expansion curves (upper right panel) at several time points. At 86 h fold expansion **(F)** of prior CD19 CAR-T cells, and IL-2 level **(G)** were measured. **(H)** live tumor cells counting after co-culture on 96 h and percentage of lysis **(F)** at 1:1, 1:5 and 1:10 ratio of E:T. *P < 0.05, **P < 0.01, ***P < 0.001 compared with the control group. Standard error means (SEM) are indicated as error bars.

By using FCM, we detected that the anti-CD19-CAR and anti-CD22-CAR were successfully expressed on T-cell surface with transduction efficiency about 77.1 and 71.2%, respectively (**Figure 1A**). Based on these results, CD19 and CD22 CAR-T cells were qualified for the following experiments. We created differential expansion curves by incubating CAR-T cells with Raji cells at E:T ratio of 3:1, 1:1 and 1:3. The number of CAR-T cells increased after 30-hour co-culture models at different ratio (**Figure 1C**), which was counted by FCM using Countbright counting beads. To evaluate the CAR-T cell expansion, prior CD19 CAR-T cells were labeled by CellTrace CFSE and co-cultured with Raji for 24 h. To be distinguished from the prior CAR-T cells, the subsequent CD19, CD22 CAR-T cells and T cells transduced with pCDH empty vector (pCDH) were labeled with CellTrace Violet and added to the culture system respectively, as well as media for the control group (**Figures 1B, D** **)**. At the following time points, the absolute number of the prior CD19 CAR-T cells labeled CFSE was measured by FCM using Countbright counting beads.

After co-culture at an E:T ratio of 1:3 for 86 h, the absolute cell numbers of prior CD19 CAR-T cells in the groups with sequentially infused with CD19 and CD22 CAR-T cells were 4.87 × 10^4^ and 4.15 × 10^4^ separately, which were significantly higher than that in the control group (2.4 × 10^4^) (P = 0.0007 and P = 0.0024, respectively; **Figure 1E**). The median expansion fold of prior CD19 CAR-T cells was much higher after sequentially infused CD19 CAR-T cells (4.15) and CD22 CAR-T cells (3.55), compared with the control group (2.06) (P = 0.0007 between CD19 CAR-T and medium, P = 0.0024 between CD22 CAR-T and medium, respectively; **Figure 1F**). A panel of cytokines in the culture supernatants was also measured at 96 h. Compared with the control group, the groups with sequential infusions of CAR-T cells had higher levels of IL-2 (**Figure 1G**). These results indicate that IL-2 produced by secondary infusion of CAR-T may promote the co-amplification of CAR-T cells.

### Enhanced Antitumor Effects After Sequential Administration of CAR-T Cells *In Vitro*

To evaluate if the addition of sequential CAR-T cells enhanced the killing capability of CAR-T cells, CD19 CAR-T cells labeled with CFSE were co-cultured with Raji at E:T ratio of 1:1, 1:5 and 1:10, then subsequent CD19, CD22 CAR-T cells labeled with CellTrace Violet or medium as control were added to the culture system at 24 h. The living tumor cells were enumerated after 96 h. For each E:T ratio, there were fewer Raji cells left in the groups with sequential CAR-T cells infusion (**Figure 1H**). Sequential infusion produced a more powerful killing activity toward the tumor cells, as demonstrated by lysis of Raji tumor cells (**Figure 1I**).

### Enhanced Antitumor Effects After Sequential Administration of CAR-T Cells *In Vivo*

**Figure 2:**
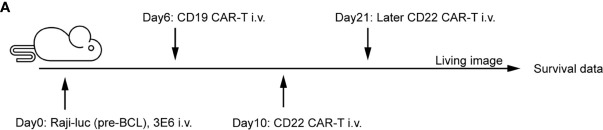
Enhanced antitumor effects after sequential administration of CAR-T cells *in vivo.* **(A)** *In vivo* treatment scheme. **(B)** Tumor burden measured by bioluminescence. **(C)** The overall survival. **(D)** The number of the prior CD19 CAR-T cells was counted before and after secondary CAR-T infusion on days 14 and 28, respectively. *P < 0.05, ***P < 0.001 compared with the control group. Standard error means (SEM) are indicated as error bars.

Our *in vitro* experiments indicated that sequential infusion of CAR-T cells are superior to single administration due to amplification of CAR-T cells and enhanced antitumor effects. The similar results were further confirmed using a xenograft model. The NSG mice received 3 × 10^6^ luciferase-expressing Raji (Raji-luc) cells *via* tail vein injection on Day 0, followed by an injection of 1 × 10^6^ prior CD19 CAR-T cells or pCDH T cells on Day 6. The mice were subsequently injected with 1×106 sequential CD22 CAR-T cells or medium on Day 10 or Day 21 (**Figure 2A**). Tumor progression was monitored by bioluminescence. The mice which received pCDH T cells or single CD19 CAR-T cells displayed faster tumor progression and became moribund at around the 3rd week after tumor inoculation. In contrast, the sequential CD22 CAR-T cell-treated mice showed milder tumor burden (**Figure 2B**). What’s more, the mice with later infusion of secondary CD22 CAR-T therapy did not show significant tumor remission (**Figure 2B**). Sequential CAR-T cell-treated mice had significantly improved survival, compared with the control groups of pCDH T cells or single CD19 CAR-T administration (**Figure 2C**). Peripheral blood from the mice was analyzed on days 14 and 28, and we observed that the absolute number of CD19 CAR-T cells in the mice with short-interval sequential infusion of CAR-T cells was significantly higher than that in the mice treated with single CAR-T cells or the long-interval sequential CAR-T cells (**Figure 2D**). However, the long-interval sequential infusion of CAR-T cells did not induce expansion of the prior CD19 CAR-T cells to reverse tumor progression. All these results demonstrated that timely sequential infusions of CAR-T cells can improve antitumor effects by enhancing the expansion of prior CAR-T cells.

### Outcomes of Short-Interval Sequential CAR-T cell Treatments in Two Advanced Patients

Two advanced patients were enrolled to receive the modified sequential therapies of CAR-T cell. Sequential CAR-T cells were infused when the number of the previous CAR-T cells in PB was detected to decreased with no severe CRS and ICANS (>Grade 3).

#### Case 1

A 15-year-old boy presented with a large mass (>10 cm) in abdominal wall. Lymph node biopsy was interpreted as BL with CD19+, CD20+, CD3+, CD10+, BCL-6+, MUM1−, BCL-2−, CD21−, CD30−, TdT−, C-MYC (80%), Ki67 (>90%), EBV-EBER (−). Fluorescence *in situ* hybridization (FISH) confirmed the only presence of MYC translocation, which excluded Double-/Triple-Hit lymphoma. Somatic TP53 mutation was detected by next generation sequencing (NGS) (21, 22). Despite having received first-line and second-line therapies including three cycles of Hyper-CVAD and two cycles of R-ICE, the patient still developed progressive disease and was diagnosed as refractory BL (High risk, III stage). It was confirmed by FCM that the tumor cells expressed antigen CD19, CD20 and CD22. Therefore, the patient received a compassionate treatment of sequential CAR-T cells.

**Figure 3:**
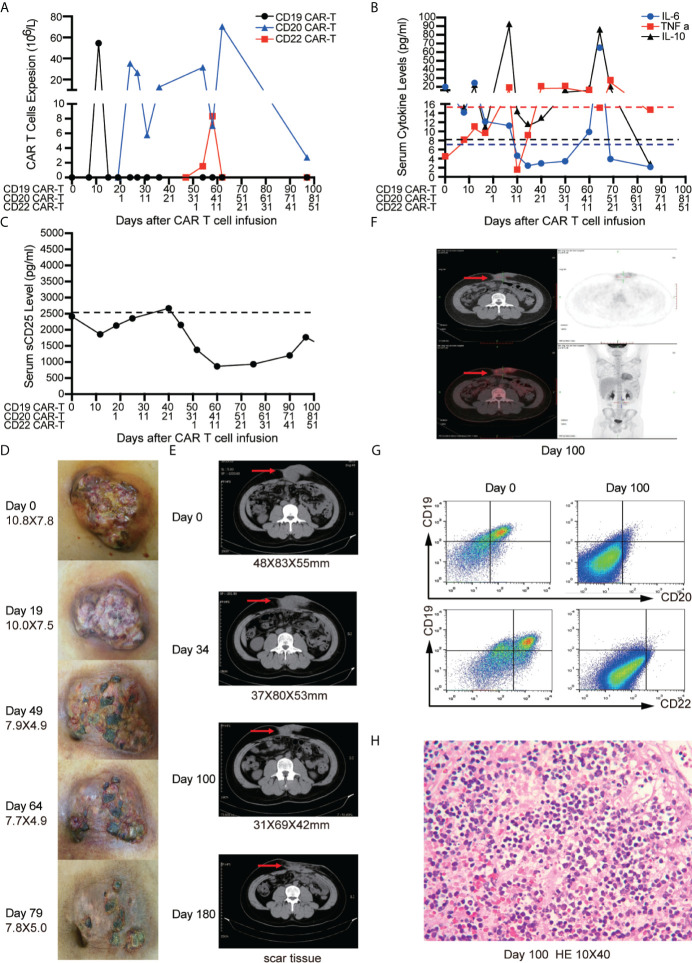
Outcomes of short-interval sequential CAR-T cell treatment in case 1. **(A)** CAR-T cell expansions during CD19, CD22, and CD20 directed CAR-T cell therapies. Detection of cytokines (IL-6, TNF-α and IL-10) **(B)** and serum soluble CD25 levels **(C)** during sequential CAR-T cell therapies. The shrinkage of tumor mass **(D)** and computed tomography (CT) images **(E)** on the timelines of sequential CAR-T cell therapies. **(F)** Position-emission tomography (PET)/CT on day 100 after CAR-T cell therapies. **(G)** FCM analysis of CD19, CD20, CD22 expression on tissue which were obtained by fine needle aspiration biopsy before and after CAR-T therapies on day 100. **(H)** Immunohistochemistry (IHC) for excisional biopsy after surgery.

After apheresis, bridging chemotherapy consisting of Aclarubicin (Acla 20 mg/days -6 to -3) and cytarabine (Ara-C 100 mg/days -6 to -3) was adopted for lymphodepletion. On day 0, CD19 CAR-T cells were infused at a total dosage of 0.8 × 106/kg. Clinical symptoms and physical signs were closely monitored. A mild fever occasionally occurred within 20 days, and the abdominal pain was not alleviated. CD19 CAR-T cells had short period of expansion in PB (only detectable on day 10) (**Figure 3A**) and the levels of cytokines including IL-6, tumor necrosis factor-α (TNF α), IL-10 (**Figure 3B**), soluble CD25 (sCD25) (**Figure 3C**) were not significantly increased (3, 21). Therefore, we conducted sequential CD20 and CD22 CAR-T cell infusions on days 19 and 49. The dosages of CD20 and CD22 CAR-T cells were 2.38 × 10^6^/kg and 1.4 × 10^6^/kg, respectively. Lymphodepleting chemotherapy was only administrated before CD20 CAR-T cell infusion. We didn’t find that CD19 CAR-T cells were induced to re-expand. The peak expansion of CD20 CAR-T cells was observed on day 27 (26.4 × 10^6^/L). With the expansion of CD22 CAR-T cells, the higher peak expansion of CD20 CAR-T cells was detected on day 62 (70.4 × 10^6^/L) (**Figure 3A**). During the sequential CAR-T cell treatment, only mild reactions (Grade I CRS) such as mild fever, tiredness, and loss of appetite were observed. In addition, the inflammatory factors (IL-6, TNF-α, IL-10) slightly increased twice with the peak expansion of CD20 and CD22 CAR-T cells (**Figure 3B**). No ICANS was observed.

The tumor mass was monitored by physical examination and CT scan. No significant shrinkage of mass was observed until day 50 (**Figures 3D, E** **)**. On day 100, Positron emission tomography/computed tomography (PET/CT) scan was performed to evaluate the therapeutic effect of CAR-T cells. The residual tumor mass didn’t display the typical malignant tumor-like hypermetabolic lesions (**Figure 3F**) and was subsequently resected. The mass tissue was examined by FCM and IHC. FCM showed that no viable tumor cells were detected in the surgical specimens (**Figure 3G**), but atrophic and necrotic cells were observed by IHC (**Figure 3H**). Due to continuous administration of entecavir, re-activation of HBV wasn’t observed, and the copies of HBV-DNA in the serum decreased from 1.8 × 10^4^ IU/ml to 2.5 × 10^2^ IU/ml.

#### Case 2

A 15-year-old girl was diagnosed as diffuse large B-cell lymphoma (DLBCL) at the IIIB stage (High risk) 2 years ago and received the first-line and second-line therapies including one cycle of COP, three cycles of CAT, one cycle of PVD-L, one cycle of VDLD, one cycle of COAD, five cycles of R-Hyper-CVAD, two cycles of R-GDP-Bortezomib-Lenalidomide and one cycle of R-EPOCH. But the patients still suffered from the progression of disease with diffused large masses (>10 cm). The biopsy of the cervical lymph nodes was interpreted as DLBCL with CD19+, CD20+, CD22+, CD3−, CD10−, CD34−, CD99−, BCL-6+, MUM1+, BCL-2+, CD30+, TdT−, C-MYC (40%), Ki67 (95%), EBV-EBER (−). Next-generation sequencing (NGS) of the resected lymph nodes revealed a TP53 mutation. FISH was negative for MYC/BCL2/BCL6 rearrangements, so Double-/Triple-Hit lymphoma was excluded. Tumor biopsy specimen showed that the tumor cells expressed antigen CD19, CD22. Therefore, she received compassionate treatment with short-interval sequential infusions of CAR-T cells which was approved by IRB.

**Figure 4:**
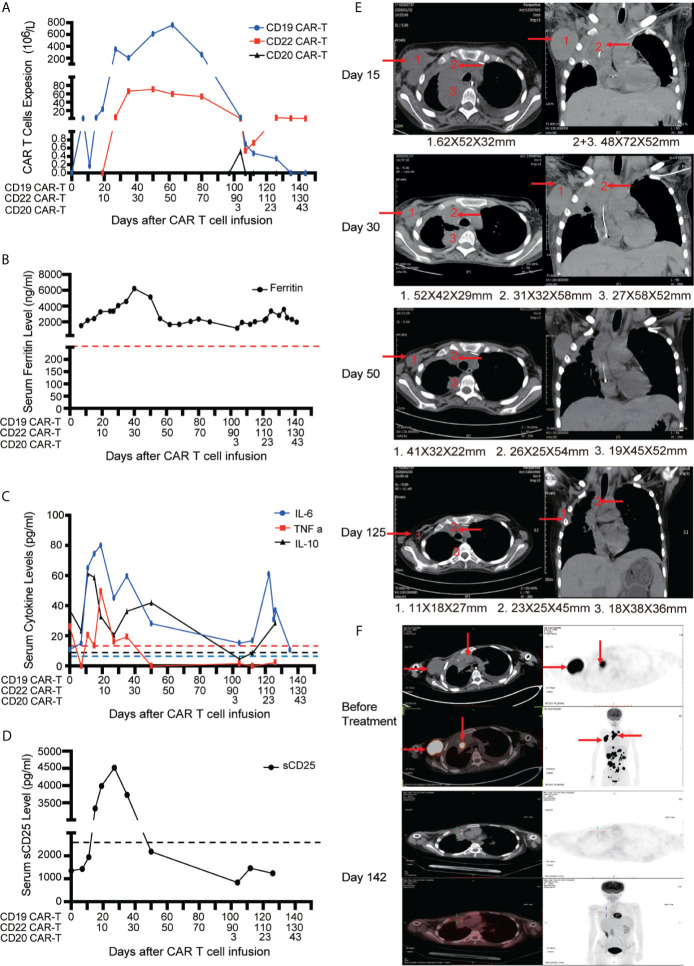
Outcomes of short-interval sequential CAR-T cell treatment in case 2. **(A)** Monitoring of CD19/CD20/CD22 CAR-T expansions in peripheral blood during sequential CAR-T cell therapies. Detection of serum ferritin level **(B)**, cytokines (IL-6, TNF-α and IL-10) **(C)** and serum soluble CD25 levels **(D)** during sequential CAR-T cell therapies. **(E)** CT images on the timelines of sequential CAR-T cell infusion. The red arrows indicate tumor sites. **(F)** Tumor lesions before and after sequential CAR-T cell therapies, detected by PET/CT.

After apheresis, the regimen of combined lymphodepleting and bridging chemotherapies (Acla + Ara-C) was given before CD19 CAR-T cell infusion because of progressive stage. Another regimen of lymphodepleting chemotherapy was only administrated before CD20 CAR-T cell infusion. On days 0 and 10, 0.5 × 10^6^/kg CD19 CAR-T cells and 0.5 × 10^6^/kg CD22 CAR-T cells were infused respectively. A very low expansion of CD19 CAR-T cells were detected in PB on day 8 with peak expanded dose of 1.31 × 10^6^/L. Delayed CD19 CAR-T cells expansion occurred with co-expansion of CD22 CAR-T cells in PB (**Figure 4A**). The higher peak expansion (760 × 10^6^/L) of CD19 CAR-T cells was observed on day 62 after the expansion of CD22 CAR-T cells. Cytokines including Ferritin, IL-6, TNFα, IL-10 and sCD25 were significantly increased accompanied with the expansions of CD19 and CD22 CAR-T cells (**Figures 4B–D**). Mild CRS (Grade 1) manifested by mild fever was observed before CD22 CAR-T cell infusion. Moderate CRS (Grade 2) manifested by persistent fever and hypoxia (requiring low-flow nasal cannula) was observed after CD22 CAR-T cell infusion, and no ICANS was observed.

The patient had dyspnea and cardiac insufficiency on day 15, which might be caused by progressive masses in right infra-axillary region and mediastinum (**Figure 4E**). Fortunately, with the enhanced antitumor effect resulted from the expansion of CD19 and CD22 CAR-T cells, the masses began to shrink on day 30. On day 50, the clinical symptoms completely disappeared. With active treatments for bleeding and necrotizing pancreatitis, intestinal feeding tube was removed and the patient started normal diet on day 60. Finally, on day 130, PET-CT assay revealed that the patient achieved CR (**Figure 4F**). Discussion

Chimeric antigen receptor (CAR) T-cell therapy emerges as a new treatment for refractory or relapsed (r/r) B-cell non-Hodgkin lymphoma (B-NHL). However, the overall response rate (ORR) is much lower in B-NHL than in r/r B acute lymphoblastic leukemia (B-ALL) patients (5–8). In this study, we hypothesize a short-interval sequential CAR-T infusion can prolong the duration of peak expansion of CAR-T cells with enhanced antitumor effects. The experiment *in vitro* indicated that the sequential addition of the CD19 and CD22 CAR-T cells significantly promoted the amplification of the prior CD19 CAR-T cells, and also contributed to tumor-killing capacity. The similar results were also demonstrated in a mouse model of aggressive B cell lymphoma. The compassionate treatments for two advanced patients further demonstrated that this modified sequential CAR-T cell therapy induced the intensive expansion of CAR-T cells and enhanced anti-tumor effect. For the sake of the immunogenicity of murine-derived CD19 CAR-T (23), humanized CD20 CAR-T or CD22 CAR-T with different scFv should be selected for the sequential infusion. Two patients with advanced B-cell lymphoma were enrolled. Despite receiving the additional bridging chemo-therapies before infusion, the patients underwent disease progression during single CD19 CAR-T cell therapy. Nevertheless, the residual CD19 CAR-T cells had a higher peak expansion peak after the infusion of the secondary CAR-T cells at a proper time. Both of the patients achieved CR because of the powerful CAR-T cell expansions and the enhanced tumor-killing capacity which resulted from the short-interval sequential infusion of CAR-T cells.

In case1, the patient endured the disease progression during CD19 CAR-T treatment, and we didn’t observe the further expansion of the residual CD19 CAR-T cells after sequential infusion of CD20 and CD22 CAR-T, which might result from the long-interval infusion of CD19 and CD22 CAR-T cells. In case 2, the subsequent CD22 CAR-T cells were administrated before the prior CD19 CAR-T cells disappeared completely, which resulted in enhanced expansion of the residual CD19 CAR-T cells along with significant tumor shrinkage. We had performed the short-interval sequential CD20 CAR-T cells infusion for the first patient following the infusion of CD22 CAR-T cells. Similar phenomenon of the amplification for prior CD22 CAR-T cells was observed, as well as reduced tumor mass. It can be seen that the time interval between sequential CAR-T cell therapies is crucial, and the sequential CAR-T cells infusion needs to be conducted before the prior CAR-T disappears. If the time interval is too long, the first CAR-T may be exhausted and cannot be augmented again.

In our previous long-interval sequential CD19-22 CAR-T cell therapy for B-ALL (16), sequential CD22 CAR-T cells were infused at the time of undetectable CD19 CAR-T cells in PB, so there was no amplification of CD19 CAR-T cells by sequentially infused CD22 CAR-T cells. CD19 CAR-T cell therapies for B-ALL achieved a high CR rate (>90%) so there is no need to further enhance CAR-T cell expansion. Nevertheless, response in B-cell lymphoma was routinely evaluated 3 months after CD19 CAR-T cell infusion in clinical trials (24), and many patients hardly achieved CR due to CAR-T cell exhaustion. Previous long-interval sequential CD19/22/20 CAR-T therapy has been used in B-NHL patient in our center (15). This strategy can improve the ORR of B-NHL with separate effect of individual CAR-T, but expansion of single CAR-T cells was not enhanced.

Previous long-interval sequential CAR-T cell infusion in B-NHL patients was conducted more than 30 days after infusion of first CAR-T cells when the patients were almost restored after CRS. Therefore, good safety profile was observed (16). In the current study, the short-interval sequential CAR-T cell infusion were conducted when the prior CAR-T cells were still detectable, which may promote cytokine production and induce CRS. Therefore, the appropriate time point for a sequential CAR-T infusion is very crucial to prevent severe CRS. Only patients with very aggressive disease that are refractory to primary CAR-T cell infusion should be strictly selected for short-interval sequential CAR-T cell therapies.

In summary, our results demonstrate that short-interval sequential infusion of different CAR-T cells can augment CAR-T cell expansion and enhance the anti-tumor effects *in vitro*, in animal models, and in two patients with advance B-cell lymphomas. For patients with very aggressive lymphoma which progresses during primary CAR-T treatment, the short-interval infusion of secondary CAR-T cells may provide an opportunity for remission. However, there were only two cases in this study, and this strategy warrants further testing in clinical trials with a large cohort of patients.

## Data Availability Statement

The raw data supporting the conclusions of this article will be made available by the authors, without undue reservation.

## Ethics Statement

The studies involving human participants were reviewed and approved by the Ethics Committee of Beijing Boren Hospital. Written informed consent to participate in this study was provided by the participants’ legal guardian/next of kin. The animal study was reviewed and approved by the Ethics Committee of Institute of Hematology, CAMS.

## Author Contributions

YM, JP, XC, XF, CL, and XX contributed to data collection, data analyses and data interpretation. BD and AC contributed to CAR-T cell manufacture and cell culture. JP, ZL, WS, JX, JD, and ZW contributed to clinical protocol. YM performed *in vitro* experiments. JP and XC directed the study. YM, XF, XC, and JP wrote the draft of the manuscript and had final responsibility to submit for publication. All authors contributed to the article and approved the submitted version.

## Funding

This work was supported by the National Key Basic Research Program of China (No. 2016YFC1303403 and No. 2016YFC1303400), the National Natural Science Foundation of China (81272325, 81760034, 81960035) and Youth Project (31501082), and the Non-Profit Central Research Institute Fund of Chinese Academy of Medical Sciences (2018PT32034 and 2019-RC-HL-013).

## Conflict of Interest

AC is also a founding member of Shanghai YaKe Biotechnology Ltd.

The remaining authors declare that the research was conducted in the absence of any commercial or financial relationships that could be construed as a potential conflict of interest.

## Supplementary Material

Supplementary Figure 1 Schematic diagram of CAR construct.**(A)** Schematic diagram of CD19/22/20 CAR construct.

Supplementary Figure 2 Sequential CAR-T infusion efficacy in mice with Raji tumor.**(A)** Tumor burden measured by bioluminescence. **(B)** The number of the prior CD19 CAR-T cells was counted before and after secondary CAR-T infusion on day 10 and day 14, respectively. **(C)** The overall survival.

Supplementary Figure 3 CAR construct.
