# Corrigendum: Short-Interval Sequential CAR-T Cell Infusion May Enhance Prior CAR-T Cell Expansion to Augment Anti-Lymphoma Response in B-NHL

**DOI:** 10.3389/fonc.2021.778039

**Published:** 2021-10-01

**Authors:** Yuan Meng, Biping Deng, Luan Rong, Chuo Li, Weiliang Song, Zhuojun Ling, Jinlong Xu, Jiajia Duan, Zelin Wang, Alex H. Chang, Xiaoming Feng, Xiujuan Xiong, Xiaoli Chen, Jing Pan

**Affiliations:** ^1^ State Key Laboratory of Experimental Hematology, National Clinical Research Center for Blood Diseases, Institute of Hematology & Blood Diseases Hospital, Chinese Academy of Medical Sciences & Peking Union Medical College, Tianjin, China; ^2^ Cytology Laboratory, Beijing Boren Hospital, Beijing, China; ^3^ Department of Hematology, Beijing Boren Hospital, Beijing, China; ^4^ Clinical Translational Research Center, Tongji University School of Medicine, Shanghai, China; ^5^ Department of Pathology, Basic Medical College of Nanchang University, Nanchang, China; ^6^ Ganzhou Key Laboratory of Molecular Medicine, The Affiliated Ganzhou Hospital of Nanchang University, Ganzhou, China; ^7^ State Key Laboratory of Experimental Hematology, Boren Clinical Translational Center, Department of Hematology, Beijing Boren Hospital, Beijing, China

**Keywords:** B-NHL, CAR-T, CD19, CD22, CD20

In the original article, there was a mistake in ***Figure 2A*** as published. **In** ***Figure 2A*** **(*in vivo* treatment scheme), the subsequent injection time was on Day 10, not on Day 14**. The corrected ***Figure 2*** appears below.

**Figure 2:**
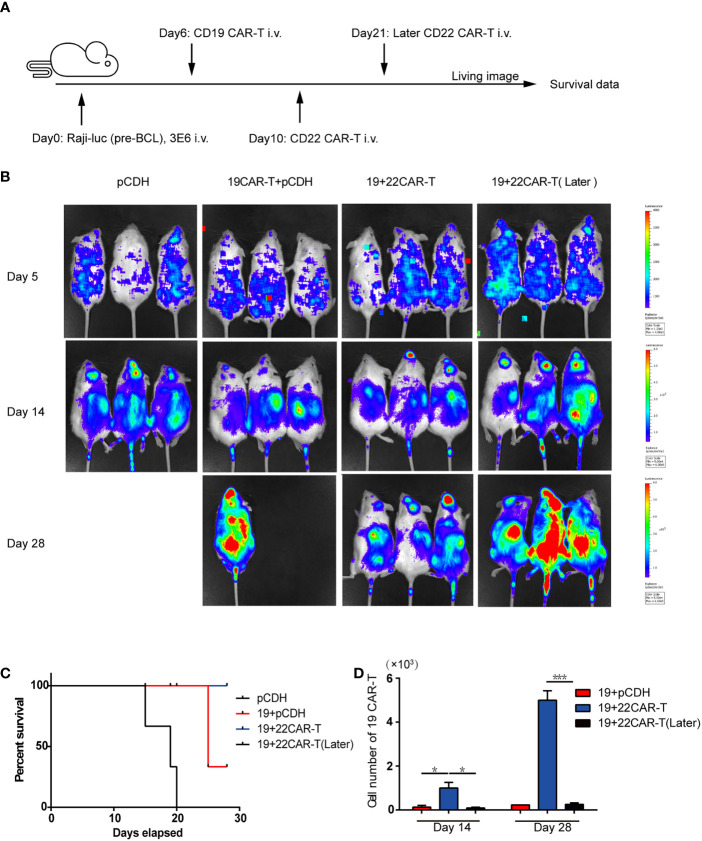
Enhanced antitumor effects after sequential administration of CAR-T cells *in vivo*. **(A)** *In vivo* treatment scheme. **(B)** Tumor burden measured by bioluminescence. **(C)** The overall survival. **(D)** The number of the prior CD19 CAR-T cells was counted before and after secondary CAR-T infusion on days 14 and 28, respectively. *P < 0.05, ***P < 0.001 compared with the control group. Standard error means (SEM) are indicated as error bars.

In the original article, there was an error. **In the *in vivo* treatment scheme, after prior CD19 CAR-T infusion, the mice were subsequently injected with 1×10^6^ sequential CD22 CAR-T cells or medium on Day 10 or Day 21, not on Day 14 or Day 21**.

A correction has been made to ***Result*, *Enhanced Antitumor Effects After Sequential Administration of CAR-T Cells In Vivo, paragraph 1*:**

“The mice were subsequently injected with 1×10^6^ sequential CD22 CAR-T cells or medium on Day 10 or Day 21 (**Figure 2A**).”

The authors apologize for these errors and state that this does not change the scientific conclusions of the article in any way. The original article has been updated.

## Publisher’s Note

All claims expressed in this article are solely those of the authors and do not necessarily represent those of their affiliated organizations, or those of the publisher, the editors and the reviewers. Any product that may be evaluated in this article, or claim that may be made by its manufacturer, is not guaranteed or endorsed by the publisher.

