# Supplementary figures and images for: Short-Interval Sequential CAR-T Cell Infusion May Enhance Prior CAR-T Cell Expansion to Augment Anti-Lymphoma Response in B-NHL
Source: Front Oncol. 2021 Jun 30;11:640166. doi: 10.3389/fonc.2021.640166 (PMC8279746; doi:10.3389/fonc.2021.640166)

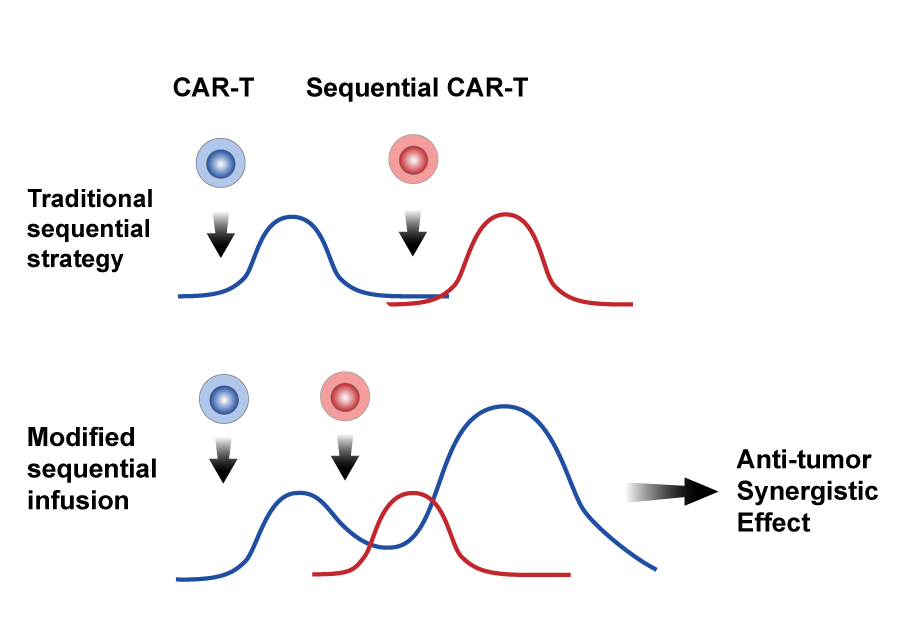

Supplement: Supplementary Figure 1 — Schematic diagram of CAR construct.(A) Schematic diagram of CD19/22/20 CAR construct. [file Image_1.tif]

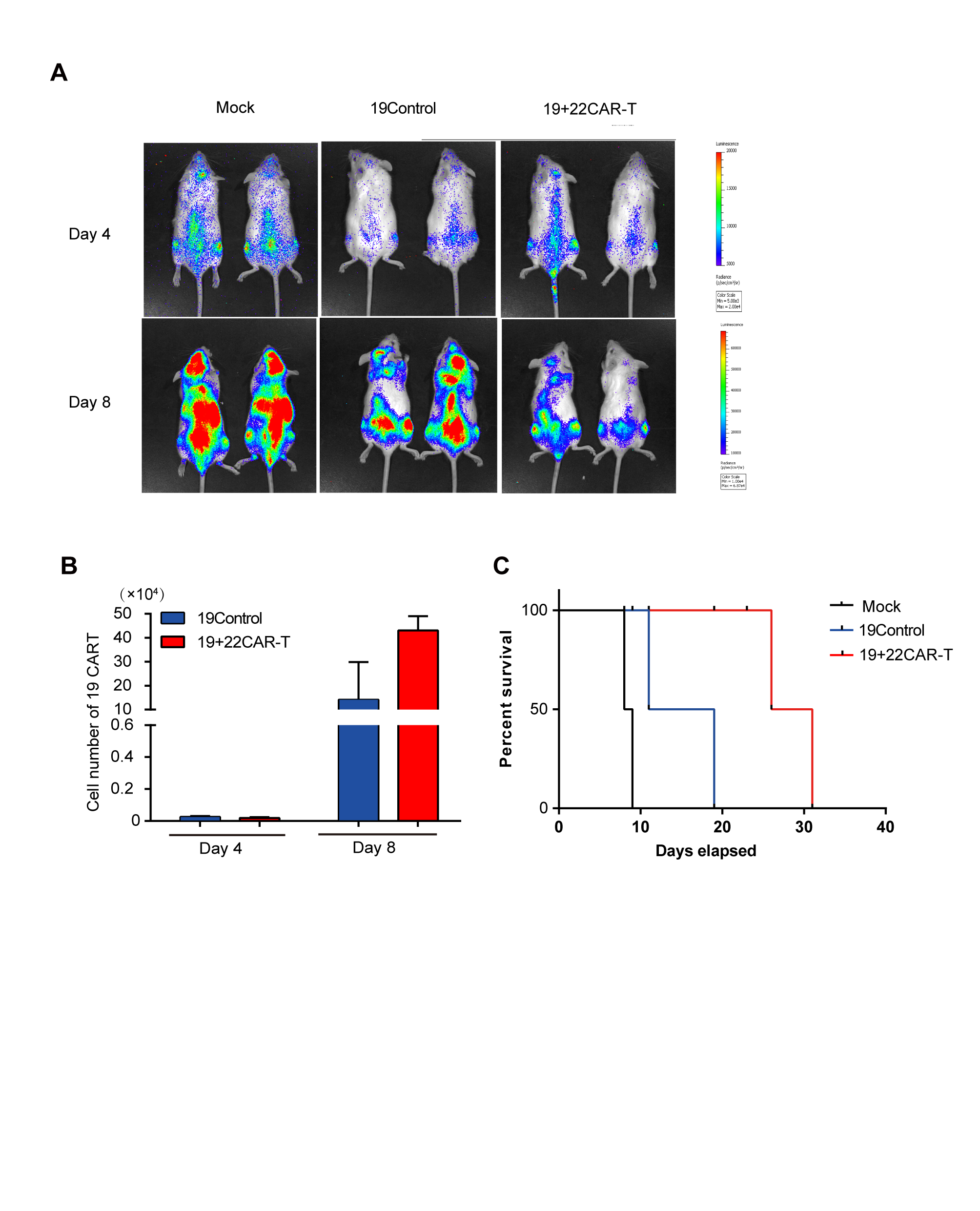

Supplement: Supplementary Figure 2 — Sequential CAR-T infusion efficacy in mice with Raji tumor.(A) Tumor burden measured by bioluminescence. (B) The number of the prior CD19 CAR-T cells was counted before and after secondary CAR-T infusion on day 10 and day 14, respectively. (C) The overall survival. [file Image_2.tif]

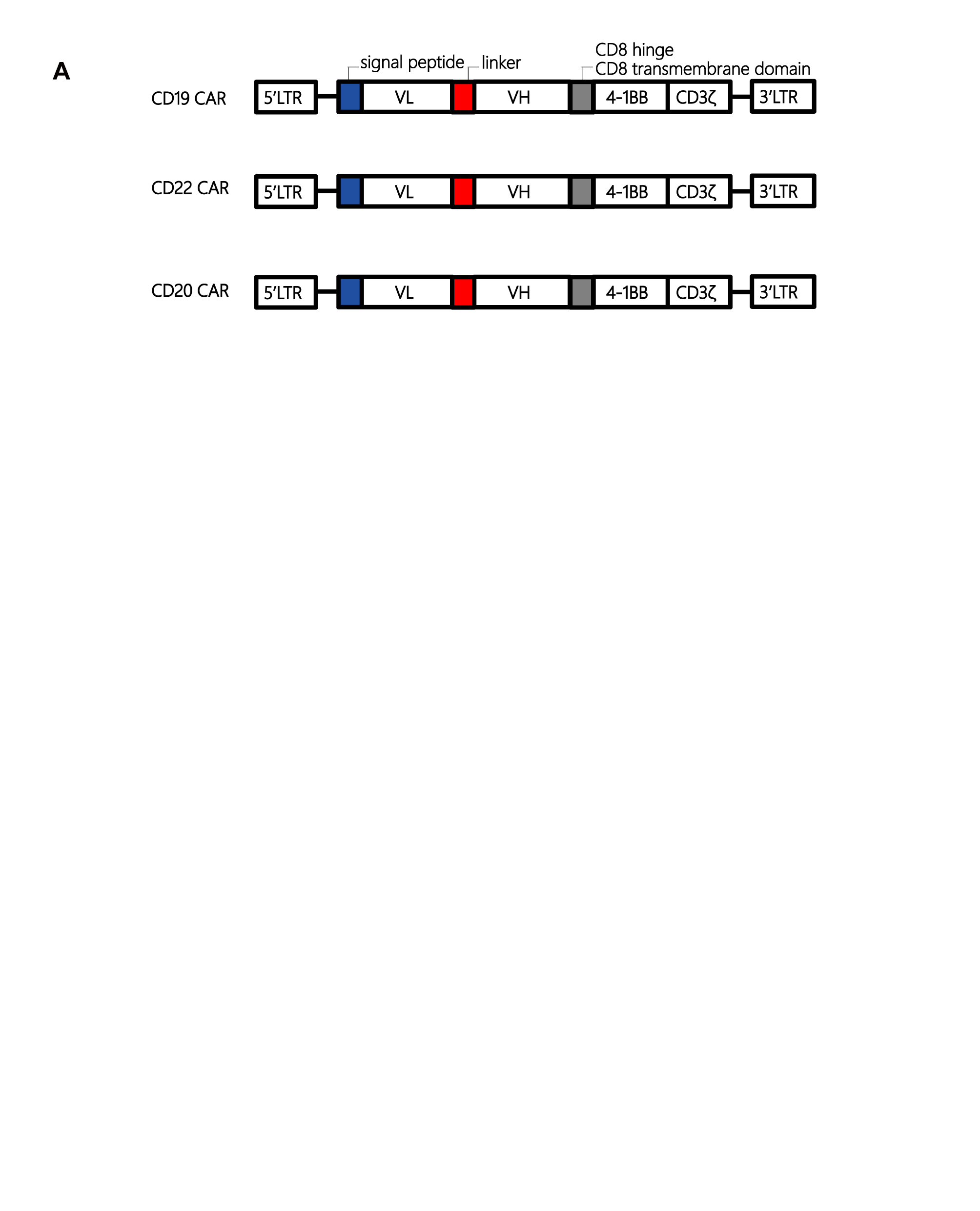

Supplement: Supplementary Figure 3 — CAR construct. [file Image_3.tif]

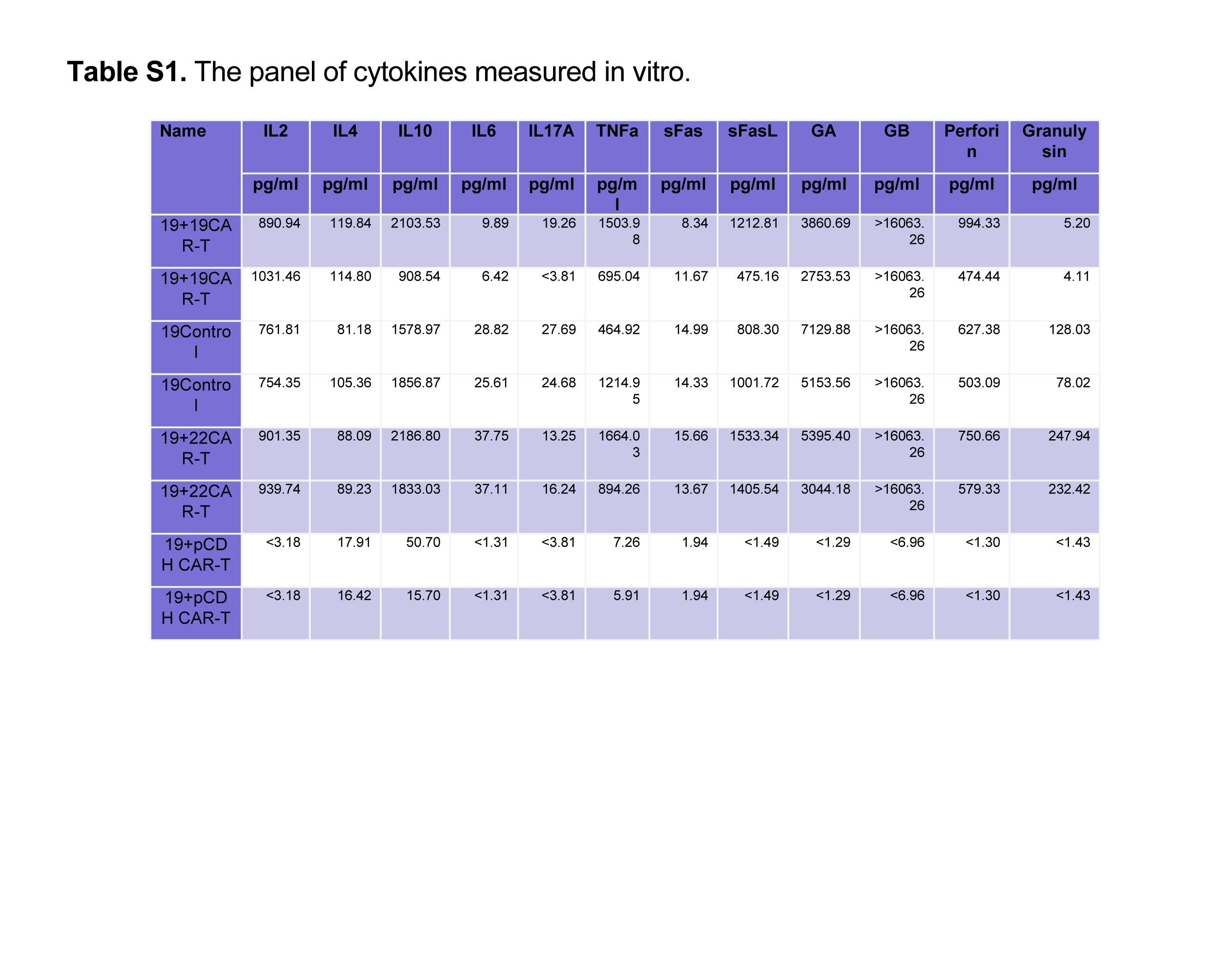

Supplement: Supplementary file 4 [file DataSheet_1.zip › supplemental Table 1.tif]
